# Estradiol Binds to Insulin and Insulin Receptor Decreasing Insulin Binding *in vitro*

**DOI:** 10.3389/fendo.2014.00118

**Published:** 2014-07-21

**Authors:** Robert Root-Bernstein, Abigail Podufaly, Patrick F. Dillon

**Affiliations:** ^1^Department of Physiology, Michigan State University, East Lansing, MI, USA; ^2^College of Osteopathic Medicine, Michigan State University, East Lansing, MI, USA

**Keywords:** polycystic ovarian syndrome, gestational diabetes mellitus, insulin resistance, sex hormone binding globulin, ovarian hyperstimulation syndrome, obesity, metabolic syndrome, molecular complementarity

## Abstract

**Rationale:** Insulin (INS) resistance associated with hyperestrogenemias occurs in gestational diabetes mellitus, polycystic ovary syndrome, ovarian hyperstimulation syndrome, estrogen therapies, metabolic syndrome, and obesity. The mechanism by which INS and estrogen interact is unknown. We hypothesize that estrogen binds directly to INS and the insulin receptor (IR) producing INS resistance.

**Objectives:** To determine the binding constants of steroid hormones to INS, the IR, and INS-like peptides derived from the IR; and to investigate the effect of estrogens on the binding of INS to its receptor.

**Methods:** Ultraviolet spectroscopy, capillary electrophoresis, and NMR demonstrated estrogen binding to INS and its receptor. Horse-radish peroxidase-linked INS was used in an ELISA-like procedure to measure the effect of estradiol on binding of INS to its receptor.

**Measurements:** Binding constants for estrogens to INS and the IR were determined by concentration-dependent spectral shifts. The effect of estradiol on INS binding to its receptor was determined by shifts in the INS binding curve.

**Main Results:** Estradiol bound to INS with a *K*_d_ of 12 × 10^−9^ M and to the IR with a *K*_d_ of 24 × 10^−9^ M, while other hormones had significantly less affinity. Twenty-two nanomolars of estradiol shifted the binding curve of INS to its receptor 0.8 log units to the right.

**Conclusion:** Estradiol concentrations in hyperestrogenemic syndromes may interfere with INS binding to its receptor producing significant INS resistance.

## Introduction

Insulin (INS) resistance associated with hyperestrogenemias occurs in gestational diabetes mellitus (GDM), polycystic ovary syndrome (PCOS), ovarian hyperstimulation syndrome (OHSS), estrogen therapies, metabolic syndrome, and obesity. The mechanism by which INS and estrogen interact is unknown, but both *in vitro* and *in vivo* studies suggest that estradiol (E2) directly or indirectly inhibits INS binding to, and/or activation of, the insulin receptor (IR).

The concentration of estrogen hormones varies across a biologically extraordinary range of almost three orders of magnitude in the normal human being, ranging between 30 pg/ml (0.06 nM) in normal males and menstruating females and 10 ng/ml (40 nM) in some pregnant women (1–7). At plasma concentrations of E2 above about 1 nM, INS resistance often develops. Some degree of INS resistance occurs in virtually all pregnant women and between 2 and 10% of pregnancies are so severely affected by GDM that they require INS treatment (5–15). Similar rates of INS resistance and diabetes occur as a result of OHSS in which E2 concentrations often exceed 10 nM (16, 17). After parturition, INS sensitivity typically returns rapidly to pre-pregnancy levels (1–10). High-dose estrogen-containing contraceptives, but not low-dose ones, have, in the past, also been associated with INS resistance and hyperinsulinemia (18–24) and in men, elevated E2 (often resulting from aromatase conversion of testosterone to E2) is also an independent risk factor for metabolic syndrome, as it may occur during puberty or following synthetic androgen use in body building (3, 25–27). The development of INS resistance also occurs in women who are treated with cyproterone acetate and ethinyl estradiol for hirsutism and/or acne (28). Administration of E2 or testosterone (which can be converted to E2) in persons undergoing sexual reassignment is another cause of INS resistance (27, 29). And 45–70% of patients suffering from PCOS have varying severities of INS resistance associated with hyperinsulinemia (30–39). In PCOS, concentrations of E2 can exceed 1 ng/ml or 4 nM (36, 37).

All of these clinical observations suggest that INS resistance is somehow related to the concentration of plasma E2.

*In vitro* experiments have helped to unravel some interactions between E2 and INS. The effects of E2 on INS activity are tri-modal. Cells that are grown in media lacking E2 or which are exposed to hypoestrogenemic conditions develop significant INS resistance as a result of decreased expression of IR and the glucose transporter GLUT4 (40–46). Normal levels of E2 are associated with normal INS and IR function. Cells exposed to hyperestrogenemic conditions develop significant decrease in INS and IR activity. Hypertestosterone syndromes such as PCOS may also contribute indirectly to E2 inhibition of INS and IR since aromatase converts testosterone to E2 resulting in hyperestrogenemia as well (30, 36, 47).

*In vivo* experiments and clinical studies confirm the *in vitro* results. Ovariectomized (OVEX) rats respond to E2 levels 100 times those found in normal gestation by decreasing the number of IR, increasing the serum concentration of INS threefold, decreasing phosphorylation of IR substrate-1, and decreasing GLUT4 expression; INS resistance ensues (48–51). Two types of data suggest that these rat studies can be extrapolated to human beings. First, functionally ovariectomized people given E2 as part of sex reassignment treatment also incur INS resistance in direct proportion to the dose of E2 (27–29). Additionally, women undergoing *in vitro* fertilization often reach supraphysiological levels of E2 >6,000 pg/ml (>10 nM) and develop INS resistance (52–59).

The combination of *in vitro* and *in vivo* and clinical studies summarized above strongly suggests that E2 alone is sufficient to induce INS resistance either directly or indirectly. Thus far, all attempts to understand E2-related INS resistance have focused on indirect mechanisms, such as reduced GLUT4 mRNA expression and transport (11, 38), increased INS synthesis and secretion (40, 60), and interactions between IR, IR complex 1 estrogen-receptor α (61). It is not clear whether these phenomena are, however, causes or results of E2-mediated activity.

We propose that a more direct mechanism may contribute to INS resistance in hyperestrogenemias: E2 may bind directly to INS and/or the IR at pathophysiologically high concentrations of E2.

## Materials and Methods

### Sex hormones

All hormones, selective estrogen-receptor modulators (SERMS) were obtained from Sigma-Aldrich: 4-andro-stenedione (A9630), cholesterol (C8667), corticosterone (C2505), estradiol (E1024), estriol (E1253), estrone (E1274), hydrocortisone (H3160), 5-Pregnen-3β-ol-20-one (P9129), progesterone (P3972), Raloxifene HCl (R1402), Tamoxifen citrate salt (T9262), and Genistein (92136). We did not test testosterone since our department lacks the approval to obtain this controlled substance.

### Peptides

Human recombinant INS (I0259 or I2643), INS A chain, oxidized (I1633) and INS B chain, and oxidized (I6383) and glucagon (G3157) were obtained from Sigma-Aldrich. Humulin R was obtained from Lilly. Peptides from the IR identified as being INS- or glucagon-like peptides (Table 1) were synthesized by the Mass Spectrometry, Synthesis, and Sequencing Facility of the Biochemistry Department of Michigan State University and purified to >98% purity as determined by high pressure liquid chromatography.

**Table 1:** **Binding constants for various sex hormone binding to recombinant human insulin (Humulin), the oxidized insulin A chain, the oxidized insulin B chain, and glucagon**.

Binding constants (*M*) at 225 nm	Humulin (human insulin)	Insulin alpha chain	Insulin beta chain	Glucagon
17β-Estradiol (E2)	1.2E−8, 6.5E−5	2.2E−04	1.5E−04	>0.0005
17α-Estradiol	1.0 E−5	>0.0005	>0.0005	>0.0005
Estriol	>0.0005	ND	ND	>0.0005
Estrone	1.25E−06	>0.0005	>0.0005	ND
Cholesterol	>0.0005	>0.0005	ND	ND
Progesterone	>0.0005	ND	ND	ND
Hydrocortisone	6.35E−05	>0.0005	>0.0005	ND
Andro-stenedione	1.35E−05	>0.0005	>0.0005	ND
Corticosterone	2.11E−05	>0.0005	>0.0005	ND
Pregnenolone	5.70E−06	>0.0005	>0.0005	ND
Raloxifene	1.3E−04	ND	ND	ND
Tamoxifen	>0.0005	ND	ND	ND
Genistein	>0.0005	ND	ND	ND

**Figure 1:**
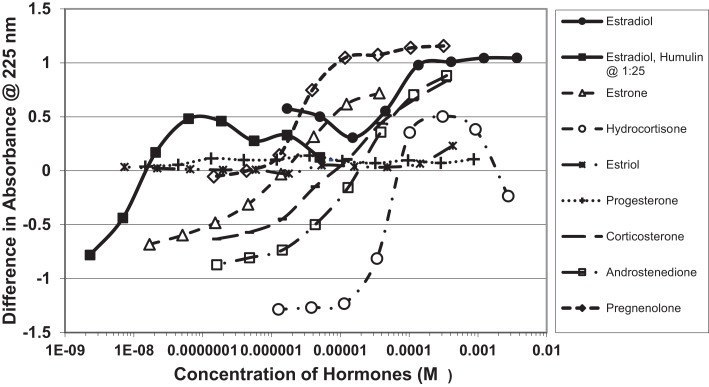
**Curves showing concentration-dependent binding of various sex hormones to recombinant human insulin (Humulin)**. Binding constants (Table 1) were approximated by determining the inflection points of these curves. Note that estradiol has two binding modes, a high affinity one that is apparent at very low concentrations of E2 (Humulin 1/25 dilution) and a lower affinity site that is present at higher concentrations of E2.

Humulin has a high affinity binding site for 17-β-estradiol (E2) as well as a low affinity site (see Figure 1). ND stands for “not done.”

### Receptors

Rat IR membrane preparation (I-9266) (IR) and human INS-like growth factor receptor (IGFR) (I-4657) were obtained from Sigma-Aldrich. The rat IR was a partially purified preparation in residual membrane and contained 250 units of activity as measured by its ability to catalyze the incorporation of 1 pmol per min of phosphate from γ-^32^P-ATP into poly(Glu, Tyr), 4:1, at 30°C. The IGFR was recombinant, at least 95% pure by SDS-PAGE, and capable of binding INS-like growth factor. Both receptor preparations, in sum, retained activity.

### UV spectrophotometry

Ultraviolet spectroscopy was used to determine binding constants of sex hormones to INS, INS peptides, IR peptides, IR, and IGFR. This method has been used for decades by other investigators (62–65) as well as by the authors of this paper (55, 56, 66–69).

Insulin receptor peptides were prepared at 100 nM in pH 7.4 phosphate buffer (Fisher Scientific). Humulin and human recombinant INS were prepared at 12.5 and 25 nM in pH 7.4 phosphate buffer. IR (250 units) and INS-like growth factor receptor (50 μg) were each prepared for assay by diluting the manufacturer’s receptor preparation in 1.0 ml pH 7.4 phosphate buffer to about 3.5 × 10^−12^ M.

Sex hormones and controls were dissolved in 95% ethyl alcohol to 1.0 mM and diluted serially by thirds.

Hormone peptide binding studies were carried out as follows. In two columns of the serially diluted steroid hormone, 100 μL of diluted peptide was added to the wells. One hundred microliters of buffer was added to the other two columns containing serially diluted steroid hormones. As controls, in a fifth column, 100 μL of the diluted peptide was added to 100 μL of 95% ethyl alcohol in two wells. To two additional wells, 100 μL of 95% ethyl alcohol and 100 μL of buffer were added. Plates were incubated at room temperature for 30 min and were then read in a SpectraMax 384Plus from 200 to 290 nm. The differences between the expected absorbance values (determined by adding the individual hormone absorbances and the individual peptide, INS absorbances minus the buffer–ethanol controls) and the observed value of the combinations were then plotted as a function of the concentration of the hormone.

An additional method of ascertaining binding involved serial additions of estrogens to INS, INS peptides, or glucagon (as a control). Peptides were dissolved as above and 200 μL added to a well in a crystal 96-well plate. Two hundred microliters of buffer was placed in an adjacent well in the plate. Five microliters of aliquots of estrogens (1.0 or 0.1 μM in 95% ethanol) or SERMS (100 μM in 95% ethanol) were then added in tandem to the peptide well and the buffer well. An additional buffer well received a 5 μL aliquot of ethanol each time an estrogen or SERM was added to any other well. Additions were made serially. The UV spectrum was obtained after each addition as above at 225 nm. In this way, all wells were diluted with ethanol at the same rate and to the same concentrations at each aliquot. The absorbance value of the buffer well after each aliquot of ethanol was subtracted from each of the other wells (peptides, hormones, or SERMS, and their combinations). The absorbance value of the peptide, glucagon, or INS at each ethanol concentration was determined; the absorbance value of each hormone (or SERM) was determined; then these individual values were added to provide an expected value of their combination in accordance with Beer’s law. The difference between this expected absorbance value after each aliquot addition and that obtained from the actual experimental combination was then determined and this difference was plotted as a function of the estrogen or SERM concentration. All binding experiments were run in duplicate and the results averaged. Binding constants were determined from the inflection point of any S-shaped curves that resulted.

This serial addition method was also used to determine the binding of estradiol to IR and IGFR.

### Capillary electrophoresis

Capillary electropherograms of INS and estrogen were prepared at a variety of voltages. Use of capillary electrophoresis to determine binding between compounds was pioneered by this laboratory (70, 71). Human recombinant INS (0.6 mg/ml; Sigma-Aldrich) and beta estradiol (1.5 mg/ml), and a combination of INS plus beta estradiol at the same concentrations, were prepared in 50 mM pH 7.4 phosphate buffer and allowed to incubate at room temperature for 48 h. The estrogen was in crystalline form and did not dissolve; only the supernatant, avoiding any estrogen crystals, was used in the experiments. Each sample was then diluted in distilled water 1:4 for a buffer concentration of 10 mM. Samples were vacuum injected into an Isco 3950 electropherograph 100 μm inner diameter glass capillary of 49 cm with a distance from injection site to detection window of 27 cm. The carrier buffer was 10 mM sodium phosphate. Samples were driven from injection site to ground using positive voltage. Each sample containing INS alone, estrogen alone, or both, was driven at 10, 15, 20, 25, and 30 kV. Peaks were detected at 200 nm wavelength.

### ^1^H nuclear magnetic resonance

Human recombinant INS (0.6 mg/ml; Sigma-Aldrich) and beta estradiol (1.5 mg/ml), and a combination of INS plus beta estradiol at the same concentrations, were prepared in 99.98% D_2_O (Sigma-Aldrich) and allowed to incubate at room temperature for 48 h. The solutions were at pH 5.5. Neither the E2 nor the INS dissolved completely, so particulates were spun down for a minute in a tabletop centrifuge and only the clear supernatant was used in the experiments. Data were collected on an Agilent Technologies 500 MHz NMR machine in the Max T. Rogers NMR Facility located in the Chemistry Department of Michigan State University. A PROTON pulse sequence (s2pul) was used at 26.0°C. The relaxation delay was 1.000 s; Pulse 45.0°; acquisition time 2.045 s; width 8012.8 Hz; 2352 repetitions. The resulting data were processed to minimize noise and to flatten the baseline.

### Enzyme-linked INS binding assay

In order to determine whether estradiol interfered with INS binding to the IR, a modified form of ELISA was used, employing INS conjugated to horse-radish peroxidase (Sigma-Aldrich) in place of an antibody (55, 56). IR was isolated from rat liver (Sigma-Aldrich). A few crystals of estradiol were added to 1.0 ml solutions of INS–HRP and IR solutions (concentrations below) and allowed to bind for 48 h. Only the supernatant of these solutions was utilized for further experimentation (i.e., only the solubilized fraction of E2). For the ELISA, the IR and IGFR (with and without E2) was utilized at 3.5 × 10^−12^ M and the INS–HRP (with and without E2) was serially diluted by thirds from an initial concentration of 50 μM. The initial concentration of E2 solubilized by the INS–HRP, INSR, and IGFR was, in each case, approximately 200 nM (see below). 100 μL of the IR or IGFR (with and without E2) was plated first and allowed to bind to the Costar EIA/RIA 96-well plate for 1 h with shaking at room temperature. The plate was then triply washed with a 1% Tween 40 solution. 200 μL of 1% polyvinyl alcohol (PVA) was added to each well and served as blocking agent. An additional set of wells were incubated with buffer alone and then the PVA to act as a control for non-specific binding in subsequent steps. The PVA was incubated and washed as described above. Next, 100 μL of the INS–HRP (with and without E2) dilutions was added to each well, incubated, and washed as above. Finally, 100 μL of 2,2′AZINO-bis (3-ethylbenziazoline-6-sulfonic acid) (ABTS single reagent, Chemicon International) was added to all wells, allowed to incubate for 30 min at room temperature, and the absorbance of each well read at 405 nm. All combinations were run in duplicate and the results averaged. Note that, because the E2 was solubilized in INS–HRP, it, too, was serially diluted as the INS–HRP was serially diluted. The concentration of E2 at the mid-point in the resulting binding curves was therefore approximately 22 nM. It is also important to note that the estradiol concentration was not directly controlled in this experiment and had to be determined by capillary electrophoretic (CE) (below). We found that use of defined solutions containing ethanol above 30% destroyed the assay.

To determine the concentration of E2 used in the experiment, a few crystals of estradiol were added to 1.0 ml of buffer containing either IR or INS–HRP and allowing these solutions to incubate at room temperature for at least 48 h. The concentration estradiol was determined using CE (pH 8.4 borate running buffer) and comparing the results to a series of known concentrations of E2 prepared in 95% ethanol. The concentration of E2 in the E2-saturated phosphate buffer in the presence of either INS or IR was found to be approximately 200 nM (ca. 60 ng/ml) or about five times the plasma concentration of E2 observed in third-trimester pregnancy.

### Data analysis

All data from the UV spectroscopy and ELISA experiments were analyzed and plotted using the Microsoft Office Excel spreadsheet program. There are no statistics or error bars because all of the methods used for determining curves and binding constants are differences in absorbance that involved multiple subtractions.

## Results

**Figure 2:**
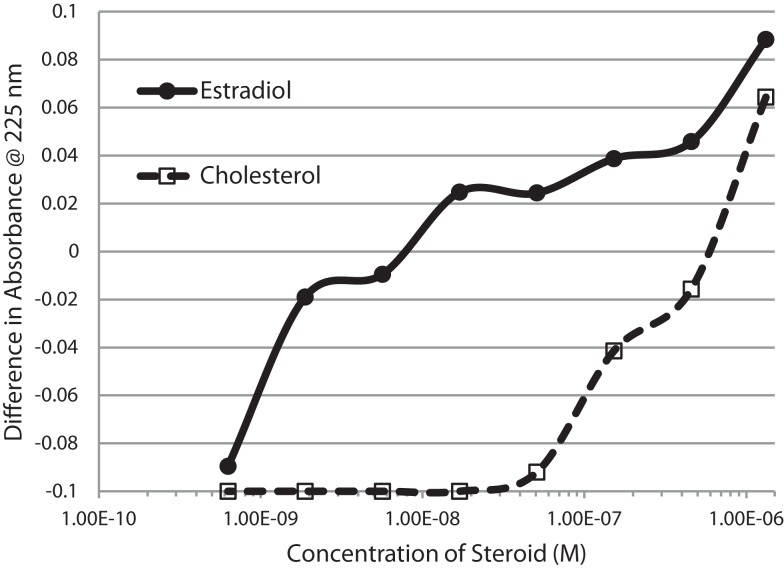
**Serial additions of estradiol (E2) also demonstrated concentration-dependent changes in the Humulin spectrum starting at nanomolar concentrations of E2**. E2 binding to cholesterol, in contrast, was observable only at micromolar concentrations. These results confirm the high affinity of E2 for insulin shown in Figure 1.

**Figure 3:**
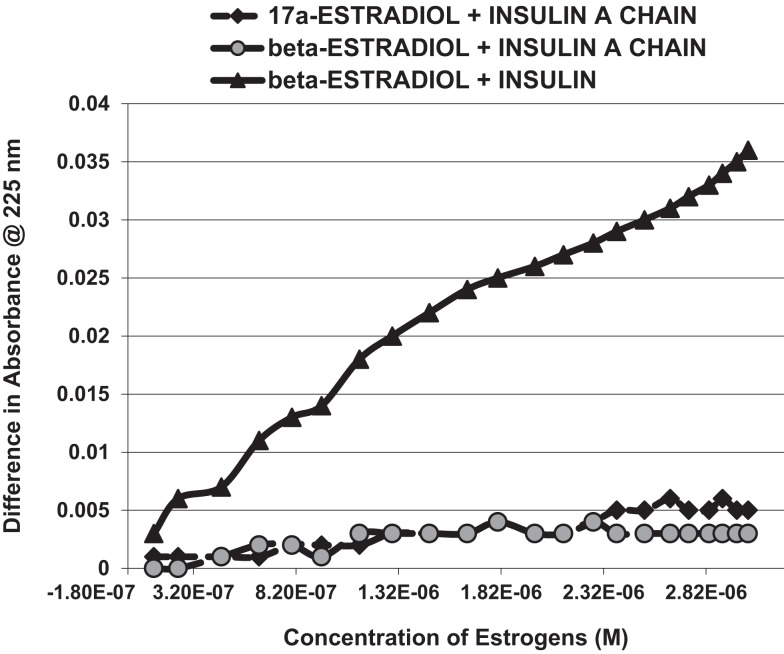
**A serial addition experiment like that shown in Figure 2, but starting with higher concentrations of Humulin and estradiols**. Even at 1000 times the concentration of E2 used in Figure 2, no binding was apparent to the insulin A chain, nor did 17-α-estradiol, an E2 antagonist, bind significantly to Humulin in this concentration range (Table 1).

**Figure 4:**
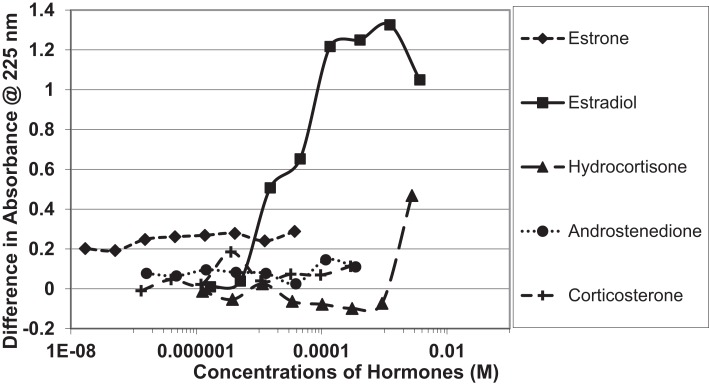
**Curves showing concentration-dependent binding of various sex hormones to the oxidized B chain of insulin**. Only estradiol (E2) showed measurable binding (see Table 1).

Figure 1 and Table 1 summarize the UV spectroscopy results of recombinant human INS binding to estradiol (E2), estriol, estrone, hydrocortisone, andro-stenedione, corticosterone, and pregnenolone and the SERM raloxifene. Estriol had a binding constant >500 μM, raloxifene 130 μM, while the other five hormones all had binding constants between 1.25 and 63.5 μM. The only hormone or SERM that bound within a physiological range was E2, which had a binding constant of 12 nM. Figure 2 confirms that measurable binding of E2 to INS is readily apparent at 10 nM, but alpha estradiol, a very weak E2 agonist, does not bind significantly to INS even at 1 μM. Binding of E2 to INS requires the cooperation of both the A and B chains of INS, since the binding constants for E2 binding to the individual oxidized chains is only 220 and 150 μM, respectively (Table 1; Figures 3 and 4). The binding of E2 to INS-like peptides derived from IR provides additional information about the specificity of the binding site. E2 does not bind with measurable affinity to glucagon (Table 1).

Notably, the binding of E2 to INS is biphasic, displaying high affinity binding (12 nM) and low affinity binding (65 μM) (Table 1; Figures 1 and 2). This biphasic binding suggests that there are at least two binding sites for E2 on INS, but the lower affinity site is unlikely to have any physiological or pharmacological importance since INS and E2 never approach concentrations even in the most extraordinary circumstances that would result in measurable binding to this site.

**Figure 5:**
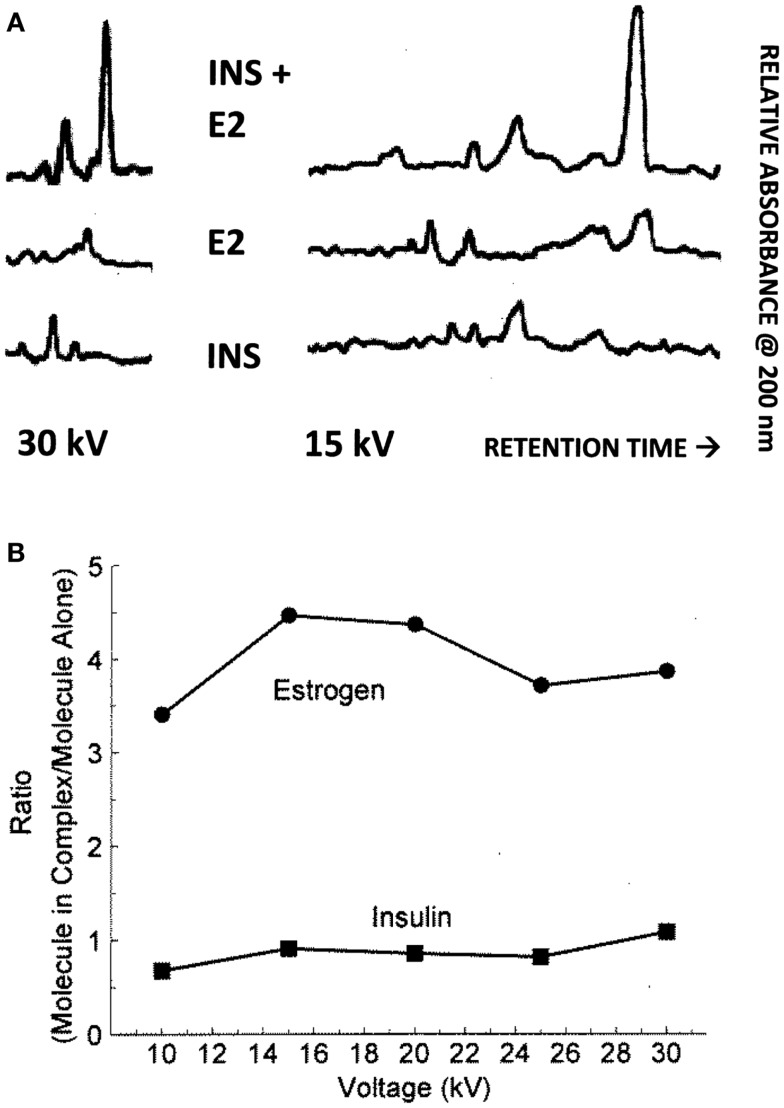
**(A)** Capillary electropherograms showing the relative absorbance at 225 nm of insulin (INS) at the bottom, estradiol (E2) in the middle, and their combination at the top, in 25 mM pH 7.4 phosphate buffer at 30 and 15 kV. Since the area of a CE peak is proportional to the concentration of the compound present, it is possible to calculate the ratio of the INS and of the E2 present in the combinations as a function of the individual samples as in **(B)**. Note that some peaks are shared in both the E2 and INS electropherograms and represent buffer material. **(B)** Illustrates the fact that while the presence of E2 has no effect on the amount of INS present in solution, the presence of INS increases the amount of E2 that is solubilized by a factor of about 4. The mean ± SD of the five EI/E ratios is 3.96 ± 0.45.

Binding of E2 to INS was confirmed by CE and ^1^H nuclear magnetic resonance spectroscopic (NMR) experiments. It is also important to know that the area under a peak representing a chemical species is, in CE, directly proportional to the concentration of the chemical species over a wide range of concentrations. Figure 5 shows the results for 15 and 30 kV experiments. Significantly, more E2 was present in solution when the E2 was mixed with INS than without. Table 2 shows that at each voltage, the peak ratio of estrogen from the estrogen/INS sample compared with the estrogen alone sample was approximately 4:1, indicating that the presence of INS causes a substantial increase in the amount of estrogen in solution. This is possible only if INS binds E2 and is consistent with the other experiments in this paper.

**Table 2:** **Binding constants for various sex hormones and insulin to peptides derived from the human insulin receptor (IR)**.

Binding constants (M) at 225 nm *peptide sequence*		Insulin-HRP	Estradiol	Estriol	Estrone	Progesterone	Andro-stenedione	Prenen-olone	Cholesterol
IR α 91–103	FRVYGLESLKDLF	**7.0E−08**	1.2E−04	>0.0005	>0.0005	>0.0005	>0.0005	>0.0005	>0.0005
IR α 105–118	LTVIRGSRLFFNY	**1.8E−09**	2.8E−04	>0.0005	>0.0005	>0.0005	>0.0005	>0.0005	>0.0005
IR α 223–234	CKSHGCTAEGLCC	>0.0005	>0.0005	>0.0005	>0.0005	>0.0005	>0.0005	>0.0005	>0.0005
IR α 233–248	CCHSECLGNCSQPDD	**8.0E−09**	>0.0005	>0.0005	>0.0005	>0.0005	>0.0005	>0.0005	>0.0005
IR α 284–300	SFCQDLHHKCKNSRRQG	**8.5E−09**	**9.0E−07**	>0.0005	>0.0005	>0.0005	>0.0005	>0.0005	>0.0005
IR α 392–404	SGYLKIRRSYALV	**3.0E−07**	>0.0005	>0.0005	>0.0005	>0.0005	>0.0005	>0.0005	>0.0005
IR α 424–441	NYSFYALDNQNLRQLWDW	>0.0005	>0.0005	3.8E−04	>0.0005	>0.0005	>0.0005	>0.0005	>0.0005
IR α 453–464	TQGKLFFHYNPK	**7.6E−09**	>0.0005	3.9E−04	>0.0005	>0.0005	>0.0005	>0.0005	>0.0005
IR β 897–916	HLCVSRKHFALERGCRLRGL	**1.5E−09**	**7.8E−07**	>0.0005	>0.0005	>0.0005	>0.0005	>0.0005	>0.0005

*Several of these peptides have previously been associated with insulin binding sites in the IR (54–56) and two of these – both insulin mimics – have significant affinity for estradiol (E2) as well. This finding is in accordance with insulin binding E2 (Table 1). Bolded figures are those that have physiological relevance*.

**Figure 6:**
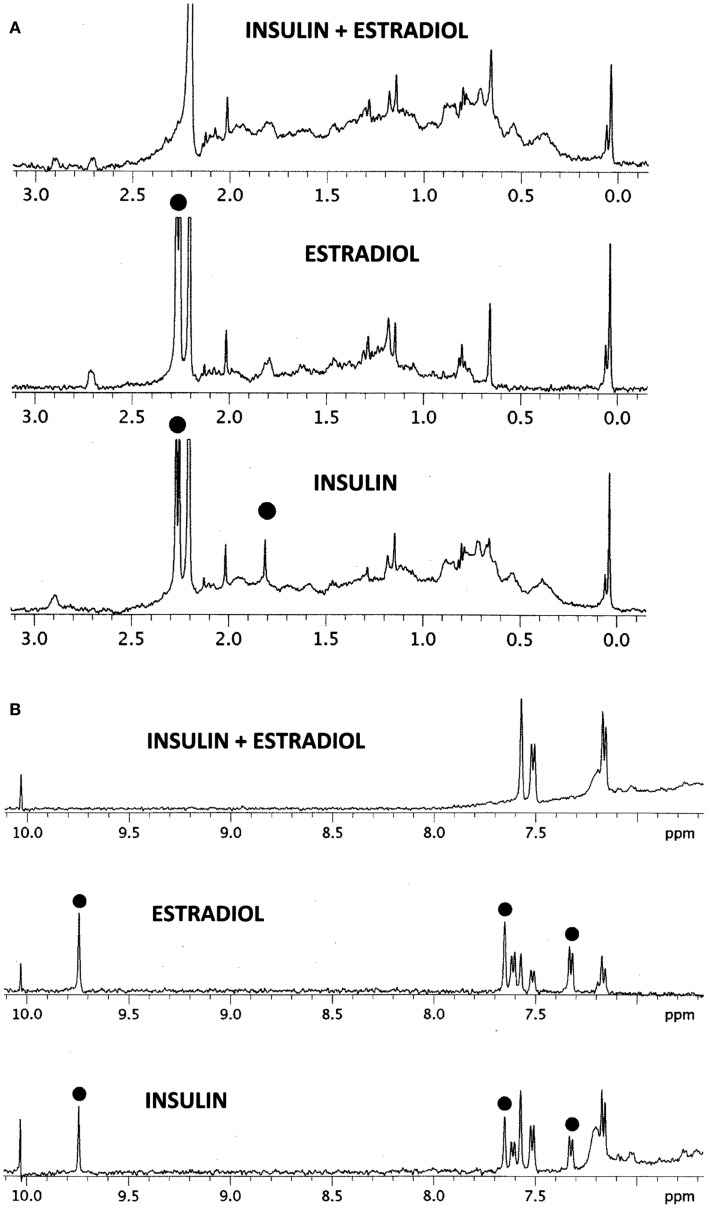
**^1^H NMR spectra in D_2_O of insulin (INS) are shown at the bottom, estradiol (E2) in the middle, and their combination (INS + E2) at the top**. **(A)** Spectral region from 0 to 3 ppm. While the various peaks of INS and E2 ranges between 0.2 and 1.5 ppm and 2.5 and 3.0 ppm add to create the INS–E2 spectrum (top), significant changes are also evident. The INS peak at 1.8 ppm (dot) broadens out in the INS–E2 combination, while the INS and E2 peaks at 2.3 ppm (dots) shift to 2.2 ppm. **(B)** Spectral region from 7 to 10 ppm. Again, some of the INS and E2 peaks are clearly additive to give the INS + E2 spectrum on top, e.g., those at 7.2 and 7.6 ppm. Other peaks in this region show major changes. The INS and E2 peaks (dots) at 9.8 ppm disappear in the combination while those at 7.35 and 7.65 ppm in both INS and E2 (dots) are quenched or shift to positions at 7.2 and 7.6 ppm. These spectral changes are consistent with E2 binding to INS involving mainly aromatic residues on both molecules.

The results of the NMR experiments, illustrated in Figures 6A,B, also indicate that E2 binds to INS. Significant changes in the spectrum occur in both the aromatic region of the spectrum, especially around 9.7 and 7.5 ppm, and also in the region of the spectrum around 2 ppm. Most strikingly, a number of peaks in both regions become so broadened or suppressed as to disappear. All such peaks are associated in both INS and E2 with aromatic hydrogens strongly suggesting that binding of E2 to INS is mediated through interactions with the histidine, phenylalanine, and/or tyrosine residues of the peptide, probably involving charge transfer complexing.

**Figure 7:**
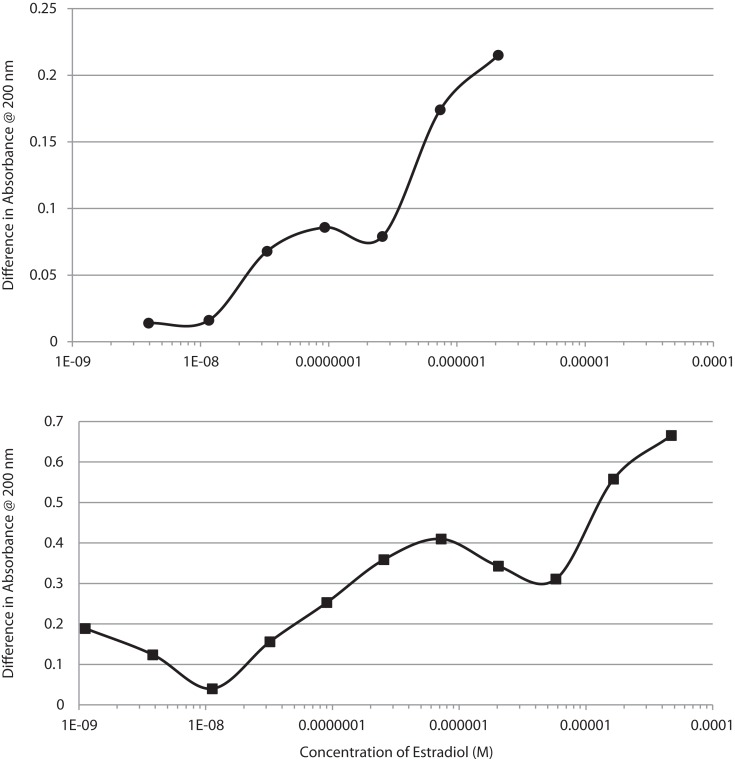
**Binding curves showing concentration-dependent effects of increasing estradiol (E2) on the UV spectrum of the insulin receptor (IR) at the top, and on the insulin-like growth factor receptor (IGFR) at the bottom**. Both curves are bimodal, showing high affinity and low affinity (probably non-specific) binding. These curves mimic those of E2 binding to insulin (Figure 1).

UV spectroscopy revealed that E2 binds to the rat IR membrane preparations with an affinity very similar to that of E2 for INS. The binding of E2 to IR was biphasic with a high affinity binding having a *K*_d_ of about 2.4 × 10^−8^ and a lower affinity binding with a *K*_d_ above 10^−6^ (Figure 7, top). E2 also bound to human recombinant IGFR, again in a biphasic manner, with high affinity binding of about 1.0 × 10^−7^ and a lower affinity binding with a *K*_d_ above 10^−5^ (Figure 7, bottom). The biphasic binding of E2 to IR and IGFR, like that of E2 to INS itself, suggests the presence of at least two binding sites for E2 on these receptors. As with INS, the low affinity E2-binding sites are extremely unlikely to play any physiological or pharmacological role.

Since we have demonstrated previously that IR and IGFR contain significant regions of homology with INS itself (53–56), the similarity between the binding constants for E2 to both INS, IR and IGFR suggested that the INS-like regions of the IR and IGFR might be the binding sites for E2. We again used UV spectrometry to investigate whether steroid hormones bind directly to INS-like peptides derived from the IR (characterization of IGFR will be reported in the future). All of the IR peptides used in these experiments are from extracellular regions of the IR, and all but one (IR peptide 897–916) are found in the alpha chain of the IR (see Table 2 for sequences and binding results). Some of the peptides tested have previously been shown to bind INS in the nanomolar range (55, 56) and are therefore likely to be involved in INS binding to the IR (see Table 2 for INS binding data). E2 bound to several of the IR peptides associated with INS binding, but at about 15- to 20-fold higher concentrations of E2 than was found for the intact rat IR preparations. E2 bound to IR peptides 284–300 (IR alpha chain) and 897–916 (IR beta chain) at 900 and 780 nM, respectively (Table 2).

**Figure 8:**
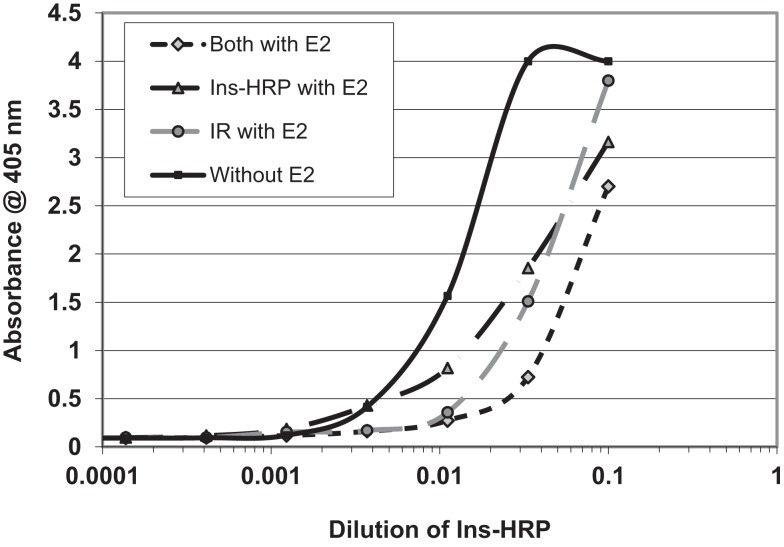
**Curves showing the effects of estradiol (E2) on the binding of insulin conjugated to horse-radish peroxidase (INS–HRP) to isolated insulin receptor (IR)**. The beginning concentration of E2 was 200 nM as measured by capillary electrophoresis (data not shown), which was diluted in tandem with the INS–HRP. The mid-point concentration of E2 in each curve was therefore about 22 nM. E2 shifted the binding curve to the right about half a log unit (a fivefold decrease in binding) when incubated with either INS–HRP or the IR alone, and it shifted the binding curve to the right 0.8 log units (an eightfold decrease in binding) when both the IR and INS–HRP were exposed to E2.

We next hypothesized that E2 binding to INS and to the IR might alter the affinity of INS for the IR. In order to explore this possibility, we used a simple enzyme-linked adsorption assay employed in previous studies of INS binding to the IR (55, 56). Figure 8 shows that the presence of E2 decreased binding of INS–HRP to the IR by about half a log unit (fivefold) when incubated with either INS or with the IR. When E2 was incubated with both INS and the IR, an eightfold decrease in INS binding to the IR resulted (Figure 6). It is important to emphasize that in each of these cases, the concentration of E2 present was initially at the maximum that INS or IR could bind in aqueous solution (200 nM E2 as determined by capillary electrophoresis) but that because the E2 was diluted in tandem with the INS and/or IR, at the inflection points of the binding curves, the concentration of E2 was about 22 nM and therefore likely represents the maximum possible effects that E2 could have under naturally occurring hyperestrogenemic conditions.

## Discussion

Figures 1–6 and Tables 1 and 2 demonstrate, using UV spectroscopy, capillary electrophoresis, and NMR, that E2 binds to INS. The UV spectroscopic studies (Figures 1–4; Tables 1 and 2) show clear E2-concentration-dependent shifts in absorbance in the nanomolar range, whereas most other estrogens, estrogen precursors, and SERMS such as raloxifene, tamoxifen, and the phytoestrogen genistein have negligible binding to INS. These data are consistent with the interpretation that E2 can cause INS resistance by direct binding to INS and IR and that the other compounds, including SERM studied here cannot produce such effects through this mechanism. In fact, SERMS are not associated clinically with development of diabetes or even INS resistance (57–59, 72–74) and phytoestrogens generally improve INS function (75–78).

The CE experiments (Figure 5) provide evidence that E2 binding to INS results in increased solubility of E2 in aqueous solution, as there is four times as much E2 in solution when INS is present as when it is absent. INS may therefore act as an E2 transporter at appropriate concentrations of the two compounds. E2-binding requires intact INS, as the oxidized INS A and B chains have very reduced affinity for E2. Comparing the INS sequence with those of the IR peptides that also bind E2 suggests that a single relatively conserved motif is necessary for E2 binding (Table 3). This motif consists of an arginine or lysine residue followed by a glycine or leucine and then a series of aromatics, usually a pair of phenylalanines and a tyrosine. At one or two amino acids removed, a hydrophilic residue such as lysine or asparagine often rounds out the motif.

**Table 3:** **Homologies among the amino acid sequences of the peptides used in the present study**.

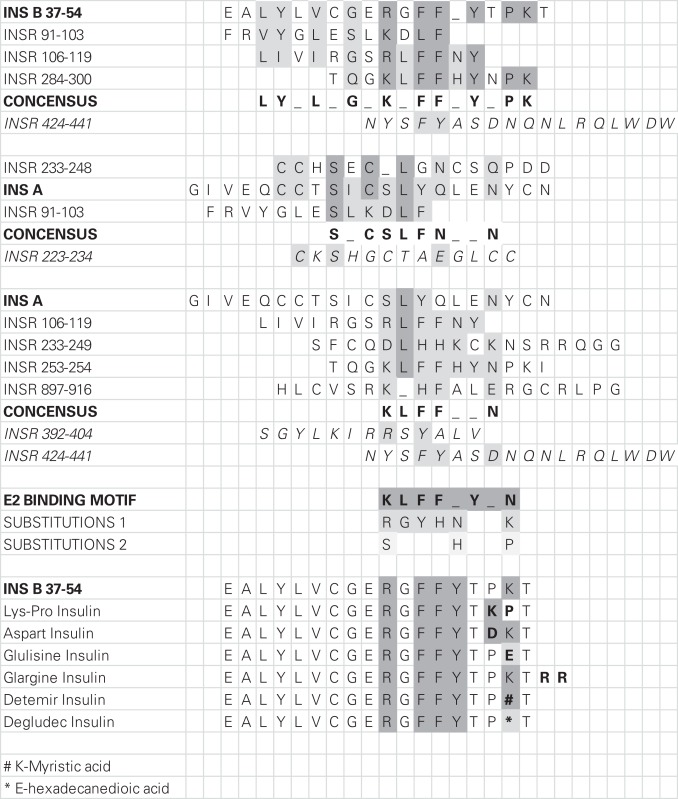

*Highly conserved residues are shown in dark shading and semi-conserved residues in light shading. Sequences that bound E2 are shown in normal font; those that did not bind E2 are shown in italicized font. The motif derived from this study is compared with a portion of the insulin B chain that is modified in many of the modified commercial insulins that are currently available*.

The NMR study (Figures 6A,B) shows major shifts and quenching of specific peaks associated with aromatic residues such as tyrosine and phenylalanine suggesting that the mechanism of binding involves pi–pi bonding or charge transfer complexing between E2 and INS. The participation of tyrosine and phenylalanine residues in E2 binding is not only logical, given the polyaromatic structure of E2 itself, but also in light of the structure of the conserved E2-binding motif derived in Table 3.

E2 binds to INS with a *K*_d_ of about 12 nM, but other steroid hormones bind to INS with significantly less affinity so that only E2 is likely to have an impact on INS activity under physiological conditions. The impact of E2 binding to INS is evaluated in Table 4. Assuming standard binding constant kinetics [*K*_d_ = (INS)(E2)/(INS–E2 complex)], it follows that at 12 nM E2, half of the INS will be complexed with E2. Concentrations of E2 exceeding 12 nM E2 are observed in a number of syndromes associated with INS resistance including third-trimester pregnancy, GDM, and OHSS, so that in these syndromes, more than half of the available INS will likely be bound to E2 at any given time, decreasing INS binding to its receptor (Figure 8). Given that over half the INS in the body would have impaired function at any given time, it would be natural for the body to “over-produce” INS to compensate. Table 3 shows that increasing the concentration of INS threefold results in about a 2.5-fold increase in free INS. At concentrations of E2 that are typical of normal males and non-pregnant females (ca. 6 × 10^−11^ M), on the other hand, complexing of E2 to INS will be negligible [on the order of two-tenths of a percent, ignoring binding of E2 to sex hormone binding globulin (SHBG), which will decrease the amount of free E2 and therefore of E2–INS as well]. Thus, the huge variation in E2 concentrations observed in human beings results in very large and very significant differences in the amounts E2 bound to INS.

**Table 4:** **Kinetic effects of estradiol (E2) binding to insulin**.

Condition	Estradiol (E2) (M)	Insulin (M)	E2–INS complex (M)	% INS bound to E2	% E2 bound to INS	% IR bound by E2
Normal male, non-pregnant female (1–3)	6 × 10^−11^	1.2 × 10^−10^	5 × 10^−13^	0.2	1.0	0.1
Luteal phase female (1, 2)	6 × 10^−10^	1.2 × 10^−10^	4 × 10^−12^	2.5	1.0	1.2
First trimester pregnancy, OHSS (3–7)	6 × 10^−9^	1.2 × 10^−10^	3 × 10^−11^	25	0.8	12
Third trimester pregnancy (3–5)	4 × 10^−8^	1.2 × 10^−10^	8 × 10^−11^	67	0.2	33
Third trimester pregnancy (3–5)	4 × 10^−8^	3.6 × 10^−10^	1 × 10^−10^	30	0.3	15
Obese type 2 diabetic (3, 54, 64, 65, 68)	2.5 × 10^−10^	3.6 × 10^−10^	7 × 10^−12^	2.1	3.0	1.0
Obese type 2 diabetic (3, 54, 64, 65, 68)	2.5 × 10^−10^	1.2 × 10^−10^	2.5 × 10^−12^	5.0	1.0	2.5

*E2 binds to insulin with an affinity of 12 nM and to the insulin receptor (IR) with an affinity of 24 nM. The table illustrates the effects of this binding as a function of various ranges of estrogen and insulin plasma concentrations. Numbers in brackets refer to references*.

As Table 3 illustrates, the impact of E2 and INS concentration variations occur between the normal baseline ranges and the extremes of pregnancy and over-production syndromes. Quadrupling E2 concentrations [which occurs frequently in obesity and is often exceeded (79)] without altering normal INS concentrations results in about 5% of the INS being bound to E2 at any given time. Compensating by tripling INS concentrations results in only 2% of the INS being bound to E2, but notably also results in 3% of the available E2 being tied up in the E2–INS complex. Each compensation, therefore, creates a negative functional effect on one or both of the hormones.

E2 binds to IR peptides as well as to INS. Tables 2 and 3 estimate the impact of E2 binding to the IR. As Table 2 demonstrates, E2 binds to INS-like peptides derived from the IR with *K*_d_ around 780–900 nM, validating the INS-dependent binding of E2 and suggesting possible locations on the IR at which E2 may bind. The binding constants for E2 binding to IR peptides reported clearly underestimate the actual binding affinity of E2 for the IR, which were determined by E2 binding to intact rat IR preparations and yielded a *K*_d_ of about 24 nM. There may be several reasons for this discrepancy. The peptides may not represent the main E2-binding sites on IR. The peptides may not be in the correct conformation for binding E2. Binding of E2 to IR may be a cooperative effect shared by several sites (as is, indeed, binding of INS to the IR). Evidence for cooperativity can be found in the fact that E2 has a much higher affinity for intact INS than for either the INS A or B chains by themselves. Finally, the binding measurements were done in solution, whereas the rat IR was membrane-bound; several studies have shown that binding of ligands to receptors improves as much as 100-fold when the receptor molecule is immobilized as the IR would be in nature (56, 67). Further investigation of the molecular determinants of E2 binding to the IR and IGFR may be warranted employing enzyme-linked E2 binding to peptides immobilized on ELISA plates, or some of the many genetically modified forms of the IR that have been produced by various laboratories in their studies of INS binding to the IR. Moreover, since the IR dimerizes with IGFR, it is possible that E2 will bind the IR:IGFR dimer with an affinity similar to the IR and IGFR affinities measured here.

E2 clearly inhibits binding of INS to IR *in vitro*. The impact of E2 binding to both INS and IR is additive, as Figure 8 demonstrates, so that the impact of increasing E2 concentrations is greater than the individual binding percentages shown in Table 3 indicated. Notably, the same effect has been observed in rats *in vivo* and *in vitro*. Hilf et al. (80) report that a 1 mg dose of E2 decreased INS binding in rats as much as 50% within 24 h while the same effect was seen in cell culture: “the addition of 10^−8^ M 17β-estradiol to the culture medium inhibited cell growth and decreased INS binding by ~20%.”

While it is clear that estradiol can produce effects on glucose regulation through a variety of indirect mechanisms, the results reported here lead us to suggest that direct binding of estradiol to INS and to the IR may be the main mechanism by which INS resistance is produced in hyperestrogenemic conditions such as gestational diabetes, OHSS and PCOS. As Table 4 illustrates, the concentrations of E2 that produce the severe INS resistance in GD, OHSS, and PCOS are in the same range as those calculated to be needed from the binding constants of E2 to INS and the IR. The fact that cell culture experiments (80), rat experiments (48–51), clinical observations of human patients undergoing sex-change therapies also demonstrate E2-concentration-dependent INS resistance or diabetes (27–29), third-trimester pregnancy (1–11), OHSS (16, 17), or PCOS (30–39, 47) are in the same concentration ranges as the *in vitro* effects suggests that the *in vitro* data can be extrapolated to *in vivo* cases.

We emphasize that direct binding of E2 to INS and IR is not the entire story behind hyperestrogenemia-associated INS resistance. Not all endogenous E2 is free in solution. It is assumed by the current literature that 80–90% of E2 is reversibly bound to SHBG, so that the effects of E2 on INS and IR may depend on the E2:SHBG ratio (12, 81). Women who develop gestational diabetes have 1.5–2.5 times higher E2:SHBG ratios than those who do not. (81). Further *in vitro* studies of how E2 is shared between SHBG, INS, and IR might clarify how this system is regulated. Other elements of the glucose regulatory and estrogen regulatory systems mentioned in the Section “Introduction,” such as GLUT and ERα expression, are certainly involved in the development of INS resistance as well. Additionally, IR activates IR complex 1 (IRC1), which interacts directly with ERα (61).

*In vivo* testing of this hypothesis will not be easy, since no direct techniques currently exist to demonstrate the binding of any hormone to another hormone or to a receptor *in vivo*. The demonstration of E2–INS and E2–IR complexes from cell cultures, animal or human plasma, or tissue samples is also fraught with difficulties, since any such experiment will require the use of purification techniques such as CE, HPLC, etc., to isolate such complexes from all of the other hormones and proteins in the original samples. Such purification is unlikely to leave complexes intact. One possible way to carry out such isolations would be to develop a form of E2 conjugated to a linker capable of cross-reacting irreversibly with any peptide or protein to which it binds. Mass spectrometry could then be used to identify the peptides or proteins to which the E2 has cross-reacted, and INS and the IR should be among those identified (in addition to, obviously, estrogen receptors). A more sensitive approach might be to create an OVEX knock-out mouse lacking estrogen receptors so that any binding to INS or IR is more readily identifiable. Development of such OVEX estrogen-receptor knockouts would also permit another type of experiment; treat the animal with radioactively labeled estradiol; then look for evidence of the accumulation of radioactivity in tissues rich in INS and IRs (e.g., the pancreas). One would expect an overlap at the microscopic level between some of the radioactivity and fluorescent antibodies against IR and INS in tissue slices from such animals.

## Conflict of Interest Statement

The authors declare that the research was conducted in the absence of any commercial or financial relationships that could be construed as a potential conflict of interest.

## Abbreviations

E2, estradiol; ERα, ERβ, alpha and beta chains of estrogen receptor; ER, estrogen receptor; GDM, gestational diabetes mellitus; INS, insulin; INS–HRP, insulin conjugated to horse-radish peroxidase; IR, insulin receptor; IVF, *in vitro* fertilization; OHSS, ovarian hyperstimulation syndrome; PCOS, polycystic ovarian syndrome; SHBG, sex hormone binding globulin.
